# Comparing the lung cancer burden of ambient particulate matter using scenarios of air quality standards versus acceptable risk levels

**DOI:** 10.1007/s00038-019-01324-y

**Published:** 2020-01-07

**Authors:** Alberto Castro, Thomas Götschi, Beat Achermann, Urs Baltensperger, Brigitte Buchmann, Denise Felber Dietrich, Alexandre Flückiger, Marianne Geiser, Brigitte Gälli Purghart, Hans Gygax, Meltem Kutlar Joss, Lara Milena Lüthi, Nicole Probst-Hensch, Peter Strähl, Nino Künzli

**Affiliations:** 1 Epidemiology, Biostatistics and Prevention Institute, University of Zurich, Hirschengraben 84, 8001 Zurich, Switzerland; 2 Formerly Swiss Federal Office for the Environment, Ittigen, Switzerland; 3 Laboratory of Atmospheric Chemistry, Paul Scherrer Institute, Villigen, Switzerland; 4 Swiss Federal Laboratories for Materials Science and Technology, Dübendorf, Switzerland; 5 Faculty of Law, University of Geneva, Geneva, Switzerland; 6 Institute of Anatomy, University of Bern, Bern, Switzerland; 7 Swiss Federal Office for the Environment, Bern, Switzerland; 8 Formerly State of Fribourg, Fribourg, Switzerland; 9 Swiss Tropical and Public Health Institute, Basel, Switzerland; 10 University of Basel, Basel, Switzerland

**Keywords:** Air pollution, Particulate matter, Lung cancer, Epidemiology, Toxicology, Health impact assessment, Carcinogens

## Abstract

**Objectives:**

Ambient particulate matter (PM) is regulated with science-based air quality standards, whereas carcinogens are regulated with a number of “acceptable” cases. Given that PM is also carcinogenic, we identify differences between approaches.

**Methods:**

We assessed the lung cancer deaths for Switzerland attributable to exposure to PM up to 10 µm (PM_10_) and to five particle-bound carcinogens. For PM_10_, we used an epidemiological approach based on relative risks with four exposure scenarios compared to two counterfactual concentrations. For carcinogens, we used a toxicological approach based on unit risks with four exposure scenarios.

**Results:**

The lung cancer burden using concentrations from 2010 was 10–14 times larger for PM_10_ than for the five carcinogens. However, the burden depends on the underlying exposure scenarios, counterfactual concentrations and number of carcinogens. All scenarios of the toxicological approach for five carcinogens result in a lower burden than the epidemiological approach for PM_10_.

**Conclusions:**

Air quality standards—promoted so far by the WHO Air Quality Guidelines—provide a more appealing framework to guide health risk-oriented clean air policymaking than frameworks based on a number of “acceptable” cases.

**Electronic supplementary material:**

The online version of this article (10.1007/s00038-019-01324-y) contains supplementary material, which is available to authorized users.

## Introduction

Ambient air pollution causes around 4.2 million annual deaths at the global level (Cohen et al. 2017). Clean air policies have been adopted by public authorities worldwide to limit exposures, and thus to minimize adverse health effects. In Switzerland, which is the focus of this study, the Swiss Environmental Protection Act (EPA) is in force since 1985 (Swiss Federal Council 2018a). It provides the legal framework for air pollution policy and entrusts the Federal Council to stipulate limit values by specific ordinances, like the Swiss Ordinance on Air Pollution Control (OAPC) (Swiss Federal Council 2018b) (see Supplementary Materials).

The regulation for ambient criteria of air pollutants such as the mass of particulate matter (PM) or some gases differs from those adopted for single carcinogenic air pollutants.

In the case of criteria pollutants, the World Health Organization (WHO) uses the global scientific literature on health effects to propose Air Quality Guidelines, which are set at a level to protect public health (WHO-Europe 2000, 2006). Epidemiological studies play a fundamental role in the assessment of the body of evidence, particularly in the setting of guideline values to prevent long-term health effects, as those are not amenable to experimental research in humans. Henceforth, we call this the “epidemiological approach.” In Switzerland, the OAPC ambient air quality standards are to a large extent in accordance with the WHO Air Quality Guidelines (WHO-Europe 2000, 2006; Swiss Federal Council 2018b).

In the case of carcinogenic air pollutants, policies acknowledge the absence of identifiable “thresholds of no effect.” Thus, given that nonzero exposures to carcinogens result in nonzero health effects, the common policy goal is to keep exposure “as low as possible” and express ambient concentrations of carcinogens in terms of risk levels, i.e., the number of cancer cases “accepted” to be caused by these pollutants. The following three risk levels are the most commonly used worldwide: 1 in 1,000,000, 1 in 100,000 and 1 in 10,000, i.e., 1 per 1,000,000, 1 per 100,000 and 1 per 10,000 persons of exposed people are expected to develop cancer due to lifetime exposure (usually defined as 70 years) to one carcinogen, respectively (WHO-Europe 2000). Henceforth, we call this the “toxicological approach” given that toxicology is often the pillar of such risk assessments. In Switzerland, an assessment commissioned by the Swiss Federal Office for the Environment (FOEN) considered 1 in 1,000,000 to most satisfactorily reflect the protection criteria as set in the Swiss EPA (Brunner 2000).

For ambient PM, the dichotomy of this risk framework is questioned twice.

First, PM is not only considered a criteria pollutant (IARC-WHO 2016, p. 36) but, since 2013, also a carcinogen (IARC-WHO 2013). The International Agency for Research on Cancer classified PM as Group 1 carcinogen (the highest risk rank), which means that there is “sufficient evidence of carcinogenicity in humans.” Exposure to ambient PM has conclusively been shown to be associated with lung cancer, while the association with other types of cancer is less certain (Loomis et al. 2013). Thus, PM is on the one side considered a complex mixture and marker of ambient air pollution—traditionally regulated with air quality standards or “limit values”—and on the other side it is a carcinogen, which is usually regulated under the “as low as possible” risk-level paradigm. In the first case, health impacts are typically derived from relative risks or excess rates published in epidemiological studies (WHO 2013) and are the basis of all estimates published by the Global Burden of Disease (Lim et al. 2012; Cohen et al. 2017). In the second case, cancer cases attributable to carcinogens are typically calculated based on their unit risks, usually derived from toxicological studies (e.g., Morello-Frosch et al. 2000; Woodruff et al. 1998).

Second, epidemiology-based risk assessments of PM now use various counterfactual concentrations. A range of previous studies (e.g., Röösli et al. 2003; Künzli et al. 1997, 2000) used a counterfactual concentration of 7.5 μg/m^3^ of PM smaller than 10 μm in aerodynamic diameter (PM_10_), reflecting the mean value of the—at that time lowest—exposure category “5–10 μg/m^3^.” Meanwhile, many epidemiological studies include participants exposed to very low outdoor concentrations of PM (Beelen et al. 2014), possibly as low as the concentrations measured at alpine monitoring stations (e.g., 2.2 μg/m^3^ for PM_10_ at Jungfraujoch in 2010). A recent study derived a novel concentration–response function for the association between long-term exposure to PM and mortality based on results from 41 cohorts conducted in 16 countries (Burnett et al. 2018). This risk function suggests the effects of PM smaller than 2.5 μm in aerodynamic diameter (PM_2.5_) to be observed down to an annual mean of 2.4 μg/m^3^. Assuming that in Switzerland PM_2.5_ accounts for 73.5% of PM_10_ (BAFU 2019), this counterfactual value is equivalent to 3.3 μg/m^3^ of PM_10_.

To date, it is not clear whether air quality standards provide equal protection of public health as the approach based on a number of “acceptable” cases. Therefore, the objective of our study was to estimate premature lung cancer deaths attributable to air pollution with both the epidemiological and the toxicological approaches and with varying choices of exposure scenarios and counterfactual concentrations for Switzerland. We focused on lung cancer mortality for both approaches given the established link with ambient PM as well as a range of single carcinogens. For the epidemiological approach, we used PM_10_ as the marker of ambient air pollution, due to the extensive availability of PM_10_ data as compared to PM_2.5_ in Switzerland. For the toxicological approach, we focused on five carcinogens: arsenic, benzo[a]pyrene (as a marker of polycyclic aromatic hydrocarbons), cadmium, elemental carbon (or soot, taken as a marker of diesel exhaust) and nickel. We restricted the assessment to inhalable particle-bound carcinogens (excluding fibers) with available unit risk factors from the literature and ambient concentration data from the National Air Pollution Monitoring Network (NABEL) (see Supplementary Materials).

## Methods

### Epidemiological approach

For the epidemiological approach, we estimated the number of premature lung cancer deaths, which are attributable to PM_10_ exposure based on the excess rate (Röösli et al. 2003) according to Eq. 1. To calculate excess rates, we applied Eq. 2 (Röösli et al. 2003).

Equation 1: Estimation of the number of lung cancer deaths by an epidemiological approach based on excess rate.

1 $$D_{\text{ER}} = \frac{{{\text{Pop}}_{ \ge 30} }}{100,000}*\left( {{\text{PWC}}_{\exp } - {\text{PWC}}_{\text{cf}} } \right)*{\text{ER}}_{1} *\left( {1 - {\text{SR}}} \right)$$

*D*_ER_ = number of lung cancer deaths that are attributable to air pollution per year based on excess rate. Pop_≥30_ = population aged 30 and older. PWC_exp_ = annual population-weighted PM_10_ mean concentration in μg/m^3^ for an exposure level. PWC_cf_ = annual population-weighted PM_10_ mean counterfactual concentration in μg/m^3^. ER_1_ = excess rate in a number of annual lung cancer cases per 100,000 persons aged 30 and older, per 1 μg/m^3^ increase. SR = survival rate of lung cancer patients.

Equation 2: Calculation of the excess rate.

2 $${\text{ER}}_{1} = I_{\text{loc}} *\ln \left( {{\text{RR}}_{{{\text{gen}},1}} } \right)$$

ER_1_ = excess rate in a number of lung cancer cases per 100,000 person-years and per 1 μg/m^3^ increase in concentration. *I*_loc_ = local observed lung cancer incidence in cases per year per 100,000 persons. RR_gen,1_ = generic relative risk of incidence (with lower and upper bounds of the 95% confidence interval from the literature) per 1 μg/m^3^ increase.

We defined four PM_10_ exposure scenarios (20, 18, 13 and 11 μg/m^3^). The first one (20 μg/m^3^) assumes the population-weighted annual mean concentration to correspond to the OPAC air quality standard (Swiss Federal Council 2018b), which is the same as the value of the WHO air quality guideline. The second exposure scenario (18 μg/m^3^) corresponds to the estimated population-weighted mean for 2010 from ten NABEL stations as derived by the FOEN and the Swiss Federal Laboratories for Material Science and Technology (BAFU 2019). In the third exposure scenario (13 μg/m^3^), we assumed that PM_10_ concentrations comply with the OPAC PM_10_ air quality standard everywhere in Switzerland, including hot spots. This is an estimate derived by FOEN and based on most recent data and spatial models. In the fourth exposure scenario (11 μg/m^3^), we assumed compliance of ambient PM_2.5_ annual mean concentrations with the newly adopted OPAC air quality standard of PM_2.5_—which corresponds to the WHO guideline value of 10 μg/m^3^—throughout the whole country. Assuming compliance with the limit value at 99% of all residential sites, the population-weighted mean concentration was 17% below the limit value, namely 8.3 μg/m^3^ of PM_2.5_ (Röösli 2014). The mean value assuming 100% compliance was not provided. Assuming that 73.5% of PM_2.5_ accounts for PM_10_ (BAFU 2019), 8.3 μg/m^3^ of PM_2.5_ can be converted into approximately 11 μg/m^3^ of PM_10_.

The health burden of these exposure scenarios was calculated against two counterfactual concentrations, namely 7.5 μg/m^3^ as annual population-weighted mean concentration of PM_10_ (Künzli et al. 2000; Röösli et al. 2003) to enable comparability with other health impact assessments and 3.3 μg/m^3^ to consider the estimation of the recently published risk function mentioned above (Burnett et al. 2018).

### Toxicological approach

For the toxicological approach, we estimated the number of lung cancer deaths caused by carcinogenic air pollutants applying Eq. 3 (Röösli et al. 2003). This equation does not account for synergistic effects between carcinogens.

Equation 3: Estimation of the number of lung cancer deaths using a toxicological approach based on unit risk.

3 $$D_{\text{UR}} = \mathop \sum \limits_{i = 1}^{n} \frac{{{\text{Pop}}_{ \ge 30} }}{100,000}*\left( {{\text{PWC}}_{i,\exp } - {\text{PWC}}_{{i,{\text{cf}}}} } \right)*\frac{{{\text{UR}}_{i} }}{\text{LT}}*\left( {1 - {\text{SR}}} \right)$$

*D*_UR_ = local number of lung cancer deaths per year that are attributable to air pollution based on unit risk. *i* = carcinogen. *n* = number of carcinogens. Pop_≥30_ = population aged 30 and older. PWC_i,exp_ = annual population-weighted mean concentration of the carcinogen *i* for an exposure level. PWC_*i*,cf_ = annual population-weighted mean counterfactual concentration of the carcinogen *i*. UR_*i*_ = unit risk in lifetime cases per 100,000 persons aged 30 and older for exposure to 1 μg/m^3^ of the carcinogen *i* (from literature). LT = lifetime in years. SR = survival rate of lung cancer patients.

The population-weighted mean concentration data of the five carcinogens are from 2010 and were provided by the FOEN (BAFU 2019). In this data set, the concentration of elemental carbon was measured as a marker of diesel exhaust at eight NABEL stations. The concentrations of the other four carcinogens were measured at ten NABEL stations (the same eight stations as for elemental carbon plus two additional ones).

When estimating the health burden, we summed the lung cancer cases under the scenarios of the three risk levels (1 in 10,000, 1 in 100,000 and 1 in 1,000,000) across the five considered carcinogens. For instance, multiplying the lifelong risk level of 1 in 1,000,000 by five carcinogens and dividing by 70 years of assumed lifetime (1*5/70) result in 0.07 deaths per 1,000,000 persons per year.

The counterfactual concentration assumes that the concentration of carcinogens is zero, since their emission is mainly due to human activity (WHO-Europe 2000; Röösli et al. 2003).

Values and references of other data used for the epidemiological and for the toxicological approach can be found in Tables 1 and 2, respectively. Further information on these data is provided in the Supplementary Materials.

## Results

For the 2010 exposure scenario, the epidemiological approach attributes 255 and 357 annual lung cancer deaths to PM_10_ in Switzerland for the counterfactual concentrations of 7.5 μg/m^3^ (scenario A2) and 3.3 μg/m^3^ (scenario B2), respectively (Table 1). This health burden is 10–14 times higher when using the epidemiological approach than when using the toxicological approach, which attributes 25 annual lung cancer deaths to the five carcinogens (scenario C1) at levels from 2010 (Table 2). Elemental carbon accounts for more than 90% of the burden of the five carcinogens included in this study.

**Table 1:** Attributable annual lung cancer deaths per 1,000,000 persons aged 30 and older and total annual lung cancer deaths in Switzerland based on the epidemiological approach adopting various exposure scenarios (including exposure in 2010) and counterfactual concentrations

Pollutant	Scenario	Ambient population-weighted mean concentration of exposure scenarios in µg/m^3a,b^	Ambient counterfactual population-weighted mean concentration in µg/m^3b^	Relative risk of lung cancer incidence per 1 µg/m^3^ PM_10_ (95% confidence interval)^c^	New lung cancer cases per year^d^	Survival rate of lung cancer^e^	Population aged 30 and older^f^	Excess rate in annual lung cancer cases per 100,000 persons aged 30 and older, per 1 μg/m^3^ (lower and upper bounds)^g^	Annual deaths per 1,000,000 persons aged 30 and older (lower and upper bounds)^g^	Annual deaths (lower and upper bounds)^g^
PM_10_	A1	20	7.5	1.006 (1.002;1.008)	4,300 (average 2011–2015)	0%	5,663,968 (state 2016)	0.429 (0.165; 0.632)	53.7 (20.6; 79)	304 (117; 448)
PM_10_	A2	18 (state 2010)	7.5	1.006 (1.002;1.008)	4,300 (average 2011–2015)	0%	5,663,968 (state 2016)	0.429 (0.165; 0.632)	45.1 (17.3; 66.4)	255 (98; 376)
PM_10_	A3	13	7.5	1.006 (1.002;1.008)	4,300 (average 2011–2015)	0%	5,663,968 (state 2016)	0.429 (0.165; 0.632)	23.6 (9.1; 34.8)	134 (51; 197)
PM_10_	A4	11	7.5	1.006 (1.002;1.008)	4,300 (average 2011–2015)	0%	5,663,968 (state 2016)	0.429 (0.165; 0.632)	15 (5.8; 22.1)	85 (33; 125)
PM_10_	B1	20	3.3	1.006 (1.002;1.008)	4,300 (average 2011–2015)	0%	5,663,968 (state 2016)	0.429 (0.165; 0.632)	71.7 (27.5; 105.6)	406 (156; 598)
PM_10_	B2	18 (state 2010)	3.3	1.006 (1.002;1.008)	4,300 (average 2011–2015)	0%	5,663,968 (state 2016)	0.429 (0.165; 0.632)	63.1 (24.2; 92.9)	357 (137; 526)
PM_10_	B3	13	3.3	1.006 (1.002;1.008)	4,300 (average 2011–2015)	0%	5,663,968 (state 2016)	0.429 (0.165; 0.632)	41.6 (16; 61.3)	236 (91; 347)
PM_10_	B4	11	3.3	1.006 (1.002;1.008)	4,300 (average 2011–2015)	0%	5,663,968 (state 2016)	0.429 (0.165; 0.632)	33.1 (12.7; 48.7)	187 (72; 276)

^a^Reference of state 2010: BAFU (2019)

^b^Details are provided in “Methods”

^c^Reference: Huang et al. (2017). The original value for 10 µg/m^3^ PM_2.5_, i.e., 1.08 (95% confidence interval: 1.03; 1.12), was converted into a value per 1 µg/m^3^ PM_10_ considering that 73.5% of PM_2.5_ concentration accounts for PM_10_ (BAFU 2019). Further details are provided in Supplementary Materials

^d^Reference: Krebsliga Schweiz (2018)

^e^Assumption

^f^67% of the total population (8,419,550) is 30 years old and older. Reference: BFS (2017)

^g^The lower and upper bounds correspond to the calculation using the lower and upper bounds of the 95% confidence interval of the relative risk

**Table 2:** Attributable annual lung cancer deaths per 1,000,000 persons aged 30 and older and total annual lung cancer deaths in Switzerland based on the toxicological approach adopting various exposure scenarios (including exposure in 2010)

Pollutant	Scenario	Ambient population-weighted mean concentration of exposure scenarios µg/m^3a^	Ambient counterfactual population-weighted mean concentration in µg/m^3b^	Geometric mean of the unit risk in annual lung cancer cases per 100,000 persons aged 30 and older, per 1 μg/m^3^ (95% confidence interval if value from multiple sources)^c^	Years of lifetime^b^	Population aged 30 and older^d^	Survival rate of lung cancer (%)^b^	Annual deaths per 1,000,000 persons aged 30 and older (lower and upper bounds)^e^	Annual deaths (lower and upper bounds)^e^	Share of deaths (%)
Arsenic	C1	3.7 × 10^−4^ (state 2010)	0	3.959 (1.015; 15.44)	70	5,663,968 (state 2016)	0	0.015 (0.004; 0.057)	0.08 (0.02; 0.32)	0.3
Benzo[a]pyrene	C1	3.0 × 10^−4^ (state 2010)	0	124.286	70	5,663,968 (state 2016)	0	0.375	2.13	8.5
Cadmium	C1	1.2 × 10^−4^ (state 2010)	0	3.928 (0.018; 854.982)	70	5,663,968 (state 2016)	0	0.005 (0; 0.986)	0.03 (0; 5.58)	0.1
Elemental carbon^f^	C1	9.4 × 10^−1^ (state 2010)	0	0.429	70	5,663,968 (state 2016)	0	4.037	22.87	91
Nickel	C1	8.6 × 10^−4^ (state 2010)	0	0.47 (0.282; 0.782)	70	5,663,968 (state 2016)	0	0.004 (0.002; 0.007)	0.02 (0.01; 0.04)	0.1
Total (5 pollutants)	C1							4.4 (4.4; 5.5)	25 (25; 31)	100
Total (5 pollutants)	C2	Lifetime risk level for each carcinogen: 1 in 10,000 (in total 5 in 10,000)			70	5,663,968 (state 2016)	0	7.14	40.5	100
Total (5 pollutants)	C3	Lifetime risk level for each carcinogen: 1 in 100,000 (in total 5 in 100,000)			70	5,663,968 (state 2016)	0	0.71	4.0	100
Total (5 pollutants)	C4	Lifetime risk level for each carcinogen: 1 in 1,000,000 (in total 5 in 1,000,000)			70	5,663,968 (state 2016)	0	0.07	0.4	100

^a^Reference: BAFU (2019). Value based on 8 NABEL stations for elemental carbon as a marker of diesel exhaust and on 10 NABEL stations (the same as for elemental carbon plus two additional ones) for arsenic, benzo[a]pyrene, cadmium and nickel

^b^Assumption

^c^References: WHO-Europe (2000), OEHHA (2009) and USEPA (2013). 95% confidence interval assumes t distribution. The unit risk of benzo[a]pyrene and elemental carbon has no confidence interval because it was available only in one review. More details are provided in the Supplementary Materials

^d^67% of the total population (8,419,550) is 30 years old and older. Reference: BFS (2017)

^e^The lower and upper bounds were derived using the bounds of the 95% confidence interval (t distribution) of the geometric mean of the unit risk factors across reviews

^f^Unit risk of “diesel exhaust,” but with elemental carbon concentration as a marker of diesel exhaust

Table 1 highlights the differences in health burdens when using different exposure scenarios and counterfactual concentrations. When comparing scenario B1 with A1, the choice of the lowest counterfactual concentration (3.3 μg/m^3^) leads to a 34% larger burden than choosing the traditional counterfactual concentration of 7.5 μg/m^3^. When comparing scenario B4 with A4, the more stringent counterfactual value results in a 120% larger burden than the one referring to the traditional counterfactual of 7.5 μg/m^3^. Further reductions of ambient PM_10_ from the estimated 2010 population-weighted mean concentration of 18–13 μg/m^3^ will reduce the attributable lung cancer deaths per year from 255 to 134 (scenario A2 vs. A3) or from 357 to 236 (scenario B2 vs. B3). Furthermore, Tables 1 and 2 show that all risk models of the toxicological approach for five carcinogens (scenario C2, C3 and C4) result in accepting much less annual lung cancer deaths in Switzerland (from 0.4 to 40.5) than any of the epidemiological scenarios for PM_10_ (from 85 to 406).

Using the epidemiological approach, we calculated the PM_10_ equivalent concentration increase, which would correspond to the three risk levels (1 in 10,000, 1 in 100,000 and 1 in 1,000,000) and to the related toxicology-based scenarios C2, C3 and C4 (5 in 10,000, 5 in 100,000 and 5 in 1,000,000, respectively) (see Supplementary Materials). If one accepts 5 lifetime lung cancer deaths per 10,000, 100,000 and 1,000,000 persons (scenarios C2, C3, C4), the population-weighted annual mean concentration of PM_10_ can be only 1.7, 0.17 and 0.017 µg/m^3^ above the counterfactual point of reference, respectively. Thus, under the most conservative risk model of 1 in 1,000,000, the PM_10_ concentrations could be only 0.003 µg/m^3^ above the counterfactual value.

As shown in our sensitivity analyses (see Supplementary Materials), all factors are similarly influential when increasing their value in both epidemiological and toxicological approaches.

## Discussion

### General findings and uncertainties

This study juxtaposes two risk assessment approaches combining four concentration scenarios with two counterfactual choices to put lung cancer deaths attributable to ambient air pollution into the context of risk assessment methods and concepts. In line with previous studies, we found that the sum of the unit risk-based attributable deaths across single carcinogens identifies only a fraction of the total burden captured with the excess rate-based epidemiological approach for PM_10_ (see Supplementary Materials). To guarantee comparability with previous assessments, we used PM_10_ instead of PM_2.5_ as the marker or air pollution (e.g., Röösli et al. 2003). In line with those studies and the Global Burden of Disease (Cohen et al. 2017), we used attributable cases instead of years of life lost (Héroux et al. 2015, 2017; Morfeld and Erren 2017).

Our quantitative comparison of the toxicology-based paradigm with the epidemiology-based assessment of attributable deaths reveals interesting differences in the (implicit) acceptance of risk underlying these two approaches. As shown in Tables 1 and 2, none of the PM_10_ scenarios fully complies with tolerating any risk level for five carcinogens.

The number of attributable deaths differs both in relative and in absolute terms under a range of alternative methodological assumptions to be discussed in more detail below.

First, our two counterfactual PM_10_ concentrations (7.5 vs. 3.3 μg/m^3^) highlight the strong influence of this parameter. Although it is appropriate to disclose attributable deaths down to very low counterfactual levels, it should be well communicated that the apparent increase in the attributable burden is caused by the alternative counterfactual value rather than by changes in the toxicity of air pollution.

Second, the values we choose for the relative risk determine the excess rate in the epidemiological approach. Ideally, the relative risk estimate would originate from Switzerland, but this is not available. We used the worldwide PM_2.5_ relative risk estimate for lung cancer incidence from the meta-analysis of Huang et al. (2017). We selected this relative risk because it is (1) from the most recent meta-analysis, (2) specific for incidence (not mixed with mortality) and (3) based on a higher number of studies than the European estimates. This choice results in a number of deaths rather similar to the one estimated in a study commissioned by the Swiss Federal Office for Spatial Development (ECOPLAN and INFRAS 2014). For public authorities, methodological consistencies facilitate the communication of results over time. However, one could also argue for other choices from the identified nine relative risk estimates published in three international meta-analyses (Raaschou-Nielsen et al. 2013; Hamra et al. 2014; Huang et al. 2017). Depending on the choice of relative risk, the attributable annual lung cancer deaths for the scenario A2 (255 in our study) range from 98 to 1079 (see Supplementary Materials). Smoking cannot explain this heterogeneity in the relative risk estimates because the studies used for the calculation of the relative risk estimate adjusted for smoking (among other factors). Whereas public authorities may prefer using the same relative risks for all consecutive studies to better compare results and trends, it is inevitable that new and possibly more appropriate risk estimates get published and, thus, used in risk assessments. Therefore, there is a need for proper communication strategies to explain the meaning of uncertainties and “conflicting results,” which are driven by methodological choices rather than by changes in the toxicity of air pollution.

Third, the choice of the lung cancer incidence impacts the excess rate. We used average incidence data from the period 2011–2015 rather than some theoretical “baseline incidence” before exposure to ambient air pollution. The latter is not available, but we conjecture this uncertainty to be of minor influence given that lung cancer incidence is most strongly driven by smoking, which tended to become less prevalent over the past decades.

Fourth, the choice of unit risk factors determines the result of the health assessment in the toxicological approach. Most unit risks are based on occupational studies (see Supplementary Materials). Transferability of the risk estimates to the general population involves uncertainties. On the one hand, this implies extrapolation of risk functions with unknown errors from much higher occupational exposures down to ambient air concentrations. On the other hand, the higher proportion of vulnerable persons in the general population or the higher toxicity of metals in acid ambient aerosols (Nordberg et al. 1985) may result in the underestimation of risks, if one relies on occupational studies alone. Similarly, the combined interaction of multiple carcinogens or between carcinogens and other pollutants is not captured in the occupational studies (Kawaguchi et al. 2006; Berenbaum 1985); thus, the health burden might be underestimated.

Fifth, the inclusion of additional carcinogens would increase the number of attributed deaths. Furthermore, some of the considered carcinogens are markers of larger groups of substances. If we had included the effect of the whole group, the resulting health burden would have been higher (see Supplementary Materials). We conclude that the restriction to five carcinogens explains part of the strong difference between the PM_10_ and carcinogen-based attributable deaths of lung cancer. PM_10_ captures not only all particle-bound carcinogens but also various interactions between these substances as well as, to some extent, interactions with correlated exposures to gases.

Sixth, derived population-weighted mean concentrations of PM_10_ and carcinogens might have some uncertainty, because they are based on a limited number of monitoring stations (up to ten in our study), but the stations are representative for most populous areas. Alternatively, PM_10_ can rely on comprehensively validated hybrid maps using spatial models, based on a range of monitoring stations, emission data and spatial information. For 2010 (scenarios A2, B2 and C1), the estimated concentration from the model was only 3% higher than the one from the NABEL stations used in our analyses; thus, our study is not sensitive to this methodological choice. A further non-quantifiable uncertainty relates to the selected year(s) to derive the exposure. Lung cancer has a long latency period, i.e., the incidence is a result of “past long-term exposure.” We used data from 2010; thus, the implicit assumption is that these values also stand for the longer-term exposure. However, the PM_10_ population-weighted concentration decreased strongly between 1991 and 2015 from over 30 to approximately 15 µg/m^3^. Similarly, concentrations of carcinogens were also reduced by varying proportions. Although the size of these temporal uncertainties is unknown, we expect all scenarios to be similarly affected; thus, comparisons across approaches and scenarios remain valid.

Seventh, we assumed that the survival rate of lung cancer cases was zero. The 10-year survival rate of Swiss lung cancer patients between 1998 and 2012 was on average 10% (11% for women and 9% for men) (Arndt et al. 2016). If we applied a nonzero survival rate, one would have obtained a proportionally lower number of attributable deaths. However, survival data for periods beyond 10 years—relevant for our risk assessment—are not available. If lung cancer is ultimately considered non-curable, our assumption may result in a negligible bias.

### Policy implications

A major motivation of this study related to the question, whether the current regulatory framework of PM, with its science-based air quality standards, remains an adequate choice despite PM now being accepted as a carcinogen. As shown in our assessment, all risk models of the toxicological approach for five carcinogens correspond to accepting much less lung cancer deaths in Switzerland than the ones attributed to PM_10_. However, although the approach to define “acceptable” cases is apparently much stricter, we see a range of advantages in maintaining air quality standards versus replacing it with the risk-level framework commonly used for single carcinogens.

First and foremost, PM_10_ is not only a carcinogen but causes a range of non-cancer morbidities and related premature deaths such as cardiovascular and respiratory diseases (WHO-Europe 2013). Furthermore, other types of cancer beyond lung cancer have been associated with PM exposure, e.g., sinonasal cancer (WHO-Europe 2000, p. 202), oral cancer (Chu et al. 2018) and possibly breast cancer (Andersen et al. 2017; White et al. 2018; Cheng et al. 2019). Indeed, the list of identified health effects of PM is constantly increasing. Under a policy framework of “acceptable” risk levels, e.g., 1 in 1,000,000, the “acceptable” target concentration would constantly change, namely decrease, with every additional outcome considered to be causally related to PM. Such “moving targets” are not only difficult to communicate to policymakers and the population at large, but also pose a major challenge for the agencies in charge of clean air development plans. In addition, “moving targets” jeopardize the proper communication of progress in clean air policy. Indeed, a policy framework defining the number of “acceptable” cases instead of setting ambient air quality standards, as used for all criteria pollutants, would force policymakers to define the number of “acceptable” cases for each air pollutant and each of the many health outcomes to then derive the related clean air target value (Thurston et al. 2017).

For carcinogens not regulated with limit values, we rather recommend agencies to continue the “as low as possible” policy. In line with this notion, the Swiss Federal Commission for Air Hygiene (EKL in German) recommended in 2013 to reduce airborne elemental carbon, as a marker of diesel exhaust, to 20% of the levels observed at that time, within 10 years (EKL 2013). Based on Table 2, this recommendation approximately corresponds to accepting around five deaths per year and it only complies with a level of risk of 1 in 1,000,000.

As shown in our assessment, air quality standards for PM provide a transparent base to estimate premature deaths under a broad range of policy scenarios. We consider of particular interest our scenario using 11 µg/m^3^ as a counterfactual PM_10_ concentration to comply with the newly adopted annual PM_2.5_ limit value. PM_10_ concentrations are substantially determined by the PM_2.5_ values, and over the past decades, clean air policies reduced ambient concentrations of both particle fractions in parallel. However, the OPAC annual air quality standards of PM_2.5_ (10 μg/m^3^) are de facto more stringent than the related PM_10_ target (20 μg/m^3^). Indeed, whereas all Swiss monitoring sites comply with the latter, PM_2.5_ concentrations remain above the limit values at several sites. Once PM_2.5_ values comply at all sites, including hot spots, the population-weighted mean PM_2.5_ is expected to be close to 8.3 μg/m^3^ and PM_10_ concentrations approximately at 11 μg/m^3^, assuming that 73.5% of PM_10_ consist of PM_2.5_ (BAFU 2019).

Our findings may also guide the upcoming revision of the WHO Air Quality Guidelines (WHO-Europe 2016), where the lack of an apparent PM threshold of no adverse effect and its definition as a carcinogen cannot be ignored either. According to the above arguments, we consider the promotion of fixed air quality guideline values appealing and appropriate. A major challenge of the WHO Air Quality Guideline does not relate to the science-based derivation of such limit values, but to globally convince governments to adopt these values in national regulations, to enforce clean air strategies (Kutlar Joss et al. 2017), to communicate health benefits of clean air policies (Henschel et al. 2012) and to provide guidance in the interpretation of the burden of ambient air pollution given its mixture of many pollutants (Héroux et al. 2015).

### Conclusions

Our comparison of the epidemiological and toxicological approach to assess the lung cancer burden in the whole population has shown that the epidemiological approach using a marker of air pollutants, e.g., PM, can better cover the exposure of the whole population than a limited selection of single carcinogenic air pollutants. Thus, applying a toxicological approach for only five inhalable particle-bound carcinogens with a risk level of 1 in 1,000,000, 1 in 100,000 and 1 in 10,000 for each carcinogen resulted in a number of lung cancer deaths that is smaller than the more comprehensive epidemiology-based derivation for PM_10_. Whereas single carcinogens may be regulated under an “acceptable” number of cases risk framework, our study emphasizes the advantage of air quality limit values to regulate complex mixtures of particulates or particle-bound pollutants such as PM, irrespective of their carcinogenicity or the absence of thresholds of no effect. Setting science-based ambient standards at a fixed level as promoted by the WHO Air Quality Guidelines remains a pragmatic, transparent and efficient tool to guide effects-oriented clean air policymaking and to monitor its success.

## Electronic supplementary material

Below is the link to the electronic supplementary material.

Supplementary material 1 (PDF 1325 kb)
