# Supplementary material for: Comparing the lung cancer burden of ambient particulate matter using scenarios of air quality standards versus acceptable risk levels
Source: Int J Public Health. 2020 Jan 7;65(2):139–48. doi: 10.1007/s00038-019-01324-y (PMC7049545; doi:10.1007/s00038-019-01324-y)
Supplement: Supplementary file 1 — Supplementary material 1 (PDF 1325 kb) [file 38_2019_1324_MOESM1_ESM.pdf]

Supplementary material

International Journal of Public Health

Article title: Comparing the lung cancer burden of ambient particulate matter using scenarios of air quality standards versus acceptable risk levels

Introduction

Swiss air pollution regulation

The Swiss EPA states in article 8 that “effects are assessed individually, collectively and according to their actions in combination”. In article 14 the Swiss EPA states that “ambient limit values for air pollution must be set such that, in the light of current scientific knowledge and experience, ambient air pollution below these levels does not endanger people, animals or plants, their biological communities and habitats, does not seriously affect the well-being of the population and does not damage buildings”. In addition, article 13 paragraph 2 of the Swiss EPA stipulates that, in setting ambient limit values, the Federal Council has to take into account the effects of pollution levels on particularly sensitive groups such as children, sick and elderly people as well as pregnant women. Article 13 paragraph 1 entrusts the Federal Council to stipulate limit values by specific ordinances.

The Swiss Ordinance on Air Pollution Control (OAPC), adopted in 1986 and updated in 2018 (Swiss Federal Council 2018), builds on the Swiss EPA and regulates aspects like the level of emissions and ambient concentrations of air pollutants (Table 1).

Table 1: Ambient limit values for air pollutants of the Swiss Ordinance on Air Pollution Control (Swiss Federal Council 2018)

Pollutant	Ambient air limit value	Statistical definition
Sulphur dioxide (SO_2)	30 µg/m^3	Annual average (arithmetic mean)
	100 µg/m^3	95% of half-hour means for one year ≤ 100 µg/m^3
	100 µg/m^3	24-hour mean; must not be exceeded more than once per year
Nitrogen dioxide (NO_2)	30 µg/m^3	Annual average (arithmetic mean)
	100 µg/m^3	95% of half-hour means for one year ≤ 100 µg/m^3
	80 µg/m^3	24-hour mean; must not be exceeded more than once per year
Carbon monoxide (CO)	8 mg/m^3	24-hour mean; must not be exceeded more than once per year
Ozone (O_3)	100 µg/m^3	98% of half-hour means for one month ≤ 100 µg/m^3

	120 μg/m³	1-hour mean; must not be exceeded more than once per year
Suspended particulates (PM_{10})	20 μg/m³	Annual average (arithmetic mean)
	50 μg/m³	24-hour mean; must not be exceeded more than 3 times per year
Suspended particulates ($PM_{2.5}$)	10 μg/m³	Annual average (arithmetic mean)
Lead (Pb) in PM_{10}	500 ng /m³	Annual average (arithmetic mean)
Cadmium (Cd) in PM_{10}	1.5 ng/m³	Annual average (arithmetic mean)
Total dust deposition	200 mg/m² * day	Annual average (arithmetic mean)
Lead (Pb) in dust fallout	100 μg/m² * day	Annual average (arithmetic mean)
Cadmium (Cd) in dust fallout	2 μg/m² * day	Annual average (arithmetic mean)
Zinc (Zn) in dust fallout	400 μg/m² * day	Annual average (arithmetic mean)
Thallium (Tl) in dust fallout	2 μg/m² * day	Annual average (arithmetic mean)

Notes: mg = milligram: 1 mg = 0.001 g, μg = microgram: 1 μg = 0.001 mg, ng = nanogram: 1 ng = 0.001 μg, PM_{10} = Particulate matter with an aerodynamic diameter of less than 10 μm. $PM_{2.5}$ = Fine particulate matter with an aerodynamic diameter of less than 2.5 μm.

Methods

Epidemiological approach

We assumed a linear exposure-response relationship (Röösli et al. 2003), as well as constant population and excess rates when comparing an exposure level with a counterfactual concentration. Furthermore, we assumed that a lung cancer case is equivalent to a lung cancer death (i.e. survival rate equals 0) and that lifetime exposure corresponds to 70 years following previous Swiss research in this field (e.g. Röösli et al. 2003). We used population data from 2016 collected by the Swiss Federal Statistical Office (BFS 2017). Following previous work, e.g. Röösli et al. (2003), we used the number of inhabitants at the age of 30 or older (5.663 million persons), since lung cancer typically occurs in this age group and most air pollution cohort studies on air pollution and lung cancer restricted the analyses on this age group, too. 67% of the total population (8,419,550) is 30 years old and older (BFS 2017).

To calculate the local incidence in Switzerland (76 new annual lung cancer cases per 100 000 persons), we divided the average number of annual new lung cancer cases in Switzerland from 2011 to 2015, i.e. 4 300 (Krebsliga Schweiz 2018), by the population of age 30 or older. As for the toxicological approach, we used Swiss population data in 2016 (BFS 2017).

We found nine different relative risk estimates from three meta-analyses (Raaschou-Nielsen et al. 2013; Hamra et al. 2014; Huang et al. 2017), which were applicable for our study (see Table 6). We selected the relative risk estimate for lung cancer prevalence due to $PM_{2.5}$ exposure from the meta-

analysis of Huang et al. (2017). The European research project European Study of Cohorts for Air Pollution Effects (ESCAPE) (Raaschou-Nielsen et al. 2013) carried out a metanalysis considering data from seven European countries (Austria, Greece, Italy, Norway, The Netherlands, United Kingdom and Sweden).. The ESCAPE study was included in two more recent meta-analysis (Huang et al. 2017; Hamra et al. 2014). Although an European estimate may be more suited for Switzerland, we selected a worldwide relative risk estimate because it is based on a higher number of studies. Huang et al. (2017) provided the relative risk estimates for lung cancer mortality and for lung cancer incidence separately, while Hamra et al. (2014) provided a common estimates mixing both concepts. Although we assumed that survival rate is zero, we decided to focus on the estimate for incidence to be consistent, since excess rate is expressed in lung cancer cases instead of deaths.

We re-scaled the relative risk estimates originally expressed as per 10 µg/m^3 increase of $PM_{2.5}$, to a value expressed as per 10 µg/m^3 increase of PM_{10} applying Equation 1 and Equation 2 . For the conversion factor between $PM_{2.5}$ and PM_{10}, we assumed that $PM_{2.5}$ accounts for 73.5% of PM_{10} concentration(BAFU 2019).

Equation 1: Re-scale of RR from a $PM_{2.5}$ to a PM_{10} form

$$RR_{PM10} = e^{ln(RR_{PM2.5})*CF}$$

RR_{PM10} = Relative risk for PM_{10} exposure
$RR_{PM2.5}$ = Relative risk for $PM_{2.5}$ exposure
CF = Conversion factor, i.e. proportion of $PM_{2.5}$ in PM_{10}

We rescaled the original relative risk ratios to per 1 µg/m^3 to enable comparability with unit risk factors applying Equation 2.

Equation 2: Re-scale of RR from a generic to a specific concentration

$$RR_s = e^{\frac{ln(RR_g)*C_s}{C_g}}$$

RR_g = Generic relative risk
RR_s = Relative risk for specific concentration
Cg = Generic concentration in µg/m^3 (normally 10)
Cs = Specific concentration in µg/m^3 (in this case 1)

Toxicological approach

Equivalent to the epidemiological approach, we used Swiss population data in 2016 (BFS 2017) and only the number of inhabitants at the age of 30 or older and we assumed that survival rate is zero.

To obtain unit risk factors of inhaled particle-bound carcinogens we consulted three large reviews conducted by the WHO Regional Office for Europe (WHO-Europe 2000), the United States Environmental Protection Agency (USEPA) (USEPA 2013) and the Office of Environmental Health Hazard Assessment of California (OEHHA) (OEHHA 2009). Thus, we identified twelve inhalable carcinogens causing lung cancer with available unit risk factors. Thereof, we considered five in our study. We included only those particle-bound substances with available concentration data in

Switzerland from NABEL stations(BAFU 2019). The five considered carcinogens are arsenic, benzo[a]pyrene, cadmium, elemental carbon and nickel. The unit risk of elemental carbon was actually derived for diesel exhaust, which is a group of substances including elemental carbon. More details on the process of selecting the carcinogens are provided below.

We focused our search of unit risk factors on inhalable particle-bound carcinogens expressed as cancer cases for lifetime exposure to a concentration of 1 μg/m^3. Carcinogens with unit risk factors expressed with a different unit, e.g. asbestos or refractory ceramic fibers (fiber/liter) were excluded from our study since optical methods are required for the measurement.

We searched the unit risk factors applying a two-step method. In a first step, we identified in three large reviews (OEHHA 2009; USEPA 2013; WHO-Europe 2000) substances, which are clearly classified as inhalable particle-bound substances causing lung cancer. In a second step, we specifically searched further information on the substances, which were found applying the above-mentioned criteria in any of the sources but not in the other(s).

The World Health Organization (WHO) Air Quality Guidelines (WHO-Europe 2000) reviews health effects of 32 ambient air pollution substances. In this report 9 substances are classified as carcinogenic based on human studies and characterized with unit risk factors expressed as estimates for lifetime exposure to a concentration of 1 μg/m3 (WHO-Europe 2000, 37). Out of them, 6 carcinogens are specifically associated to lung cancer: acrylonitrile, arsenic, benzo[a]pyrene, chromium (VI), nickel and trichloroethylene.

The Integrated Risk Information System (IRIS) of the United States Environmental Protection Agency (USEPA) (USEPA 2013) contains health effects of around 500 substances, including 65 inhaled carcinogens. We selected in the search engine of IRIS the following filter criteria: a) carcinogenic b) inhaled c) associated with respiratory-related cancer types and d) available unit risk as estimate for lifetime exposure to a concentration of 1 μg/m3. The search provided 21 substances. Out of them 6 were specifically associated to lung cancer: inorganic arsenic, beryllium and compounds, cadmium, chromium (VI), nickel subsulfide and nickel refinery dust. Nickel subsulfide is a component of nickel refinery dust, thus the average unit risk was considered for nickel as a single carcinogen.

A report of the California Environmental Protection Agency (OEHHA 2009) identifies 23 air pollution carcinogens. Out of them, we found 10 substances causing lung cancer: acrylonitrile, inorganic arsenic, benzo[a]pyrene, beryllium, bis(chloromethyl)ether (BCME), 1,3-butadiene, cadmium, chromium (VI), diesel exhaust, nickel, trichloroethylene and vinyl chloride.

In the second search step, i.e. when searching further information of substances that were identified in any of the sources but on in the other(s), we found three additional unit risk estimates. Thus, acrylonitrile and bis(chloromethyl)ether (BCME), which were generically labelled as “respiratory cancer” in the USEPA data set, were double-checked and we found that they were

specifically associated to lung cancer in the specific USEPA documentation. Moreover, Acrylonitrile was classified by the OEHHA as causing “human respiratory tract cancer” but a closer look into the documentation showed that this substance is specifically associated with lung cancer. After consulting the three reviews with the two-step search, we found in total unit risk factors for 12 carcinogens (Table 2).

For a number of carcinogens, the unit risk factors are available only in some of the sources (and not all) because of the following reasons.

- Benzo[a]pyrene was classified in the OEHHA report as causing “male hamster respiratory tract tumor”, but lung tumors were absent in the considered studies. The USEPA labelled Benzo[a]pyrene as cause of gastrointestinal and respiratory cancer and a unit risk factor is provided. However, there is limited evidence of the association with lung cancer (“there is considerable support for an association between benzo[a]pyrene exposure and lung cancer, although the relative contributions of benzo[a]pyrene and of other PAHs cannot be established”).
- No information about beryllium is provided by the WHO report.
- No information about bis(chloromethyl)ether (BCME) is provided by the WHO report.
- 1-3 Butadiene was classified as multisite carcinogen by the WHO, but no unit risk was provided due to the high variation of values across studies. The USEPA provides a unit risk factors, but only acknowledge the association with leukemia and not with lung cancer.
- Cadmium was classified by the WHO as carcinogenic but due the influence of concomitant exposure in the considered studies no unit risk was provided for lung cancer.
- Diesel exhaust was not identified in the first search in the USEPA’s data set because it had no label for tumor site. In a second search we found that it is “likely to be carcinogenic”, i.e. there is “strong but not sufficient evidence” of the association between exposure to diesel exhaust and lung cancer. Therefore, no unit risk was provided by the USEPA. No information about diesel exhaust is provided by the WHO report.
- Trichloroethylene is considered as carcinogenic by the USEPA, but only in the association with hematologic, hepatic, and urinary tumors.
- Vinyl chloride is considered as carcinogenic by the WHO and the USEPA but only the association with liver tumors is acknowledged.

Table 2: Weight of evidence (IARC-WHO 2018; WHO-Europe 2000; USEPA 2013) and unit risk factors (OEHHA 2009; USEPA 2013; WHO-Europe 2000) for lung cancer of inhalable carcinogens

**Inhalable substance causing lung cancer**	**Weight of cancer evidence**		**Unit risk in lung cancer cases per 100,000 persons for 1µg/m3 concentration of carcinogen and for a lifetime**					
	**WHO[a][b]**	**USEPA[c][d]**	**WHO[b]**		**USEPA[d]**		**OEHHA[e]**	
			**Value**	**Type of study[f]**	**Value**	**Type of study[f]**	**Value**	**Type of study[f]**
Acrylonitrile	2B	B1	2	A	6.8[h]	HO	29[h]	HO
Arsenic	1	A	150	HO	430	HO	330	HO
Benzo[a]pyrene	1	CH	8,700	HO				
Beryllium	1	B1			240	HO	240	HO
Bis(chloromethyl)ether (BCME)	1	A			6,200[h]	A	1,300	HO
1,3-Butadiene	2A						17	HO
Cadmium	1	LH			180	HO	420	HO
Chromium (VI)	1	A	4,000	HO	1,200	HO	15,000	HO
Diesel exhaust	1	LH					30	HO
Nickel[g]	1	A	38	HO	36	HO	26	HO
Trichloroethylene	1		0.04	A			0.2	HO
Vinyl Chloride							7.8	HO

[a] IARC = According to the International Agency for Research on Cancer

[b] IARC classification: 1 = Carcinogenic to humans, 2A = Probably carcinogenic to humans (proven human carcinogens, and carcinogens with at least limited evidence of human carcinogenicity, 2B = Possibly carcinogenic to humans (inadequate evidence in humans but sufficient evidence in animals), 3 = not classifiable, 4 = probably not carcinogenic.

[c] USEPA = According to the United States Environmental Protection Agency

[d] USEPA 1986 classification: A = Human carcinogen, B1 = Probable human carcinogen (based on limited evidence of carcinogenicity in humans), B2 = Probable human carcinogen (based on sufficient evidence of carcinogenicity in animals), C = possible human carcinogen, D = not classifiable; E = evidence of non-carcinogenicity. USEPA 2005 Classification: CH = carcinogenic to humans, LH = likely to be carcinogenic, SE = suggestive evidence of carcinogenic potential, InI= inadequate information to assess carcinogenic potential, NH = not likely to be carcinogen.

[e] OEHHA = According to the California Environmental Protection Agency, Office of Environmental Health Hazard Assessment

[f] A = Only based on animal studies. HO = Based on human studies but only with occupational exposed. HG = Based on studies and with general population involved.

[g] USEPA differentiates between nickel refinery dust and nickel subsulfide. We assumed the average unit risk among both substances.

[h] We found this unit risk after looking at further documentation in a second search step, i.e. after identifying that this unit risk factor was missing for this source but available in other source(s).

Out of the twelve identified inhalable substances causing lung cancer, seven are particle-bound, i.e. not volatile. We considered the substances as volatile if the boiling point was below 240ºC (NIH 2019), following the Swiss Ordinance on the Incentive Tax on Volatile Organic Compounds (Swiss Federal Council 1997). For five of the particle-bound substances, we found concentration data in

Switzerland from National Air Pollution Monitoring Network (NABEL) stations (BAFU 2019). These five carcinogens were considered for our study, namely, arsenic, benzo[a]pyrene (as marker of polycyclic aromatic hydrocarbons), cadmium, elemental carbon (as marker of diesel exhaust) and nickel.

Table 3 Selection of carcinogens for the study based on availability of unit risk, solid state and availability of concentration data of the substances for Switzerland.

Inhalable substance causing lung cancer with available unit risk	Particle-bound substance (boiling point above 240ºC)	Population-weighted mean concentration in Switzerland from National Air Pollution Monitoring Network (NABEL) stations (μg/m^3)	Carcinogens considered for this study (solid & available concentration data)
Acrylonitrile	No		No
Arsenic	Yes	3.7x10^{-4} in 2010 (BAFU 2019)	Yes
Benzo[a]pyrene	Yes	3.0x10^{-4} in 2010 (BAFU 2019)	Yes
Beryllium	Yes	No data	No
Bis(chloromethyl)ether (BCME)	No		No
1,3-Butadiene	No		No
Cadmium	Yes	1.2x10^{-4} in 2010 (BAFU 2019)	Yes
Chromium (VI)	Yes	No data	No
Elemental carbon[a]	Yes	9.4x10^{-1} in 2010 (BAFU 2019)	Yes
Nickel	Yes	8.6x10^{-4} in 2010 (BAFU 2019)	Yes
Trichloroethylene	No		No
Vinyl Chloride	No		No

[a] The unit risk estimate was derived for diesel exhaust, which is a group of substances including elemental carbon, while the concentration data are for elemental carbon as marker of diesel exhaust

For three of the five carcinogens considered the unit risk factor was available in more than one large review. We calculated the unit risk factors of the carcinogens by obtaining the geometric mean of the values reported in these reviews and the 95% confidence interval assuming t distribution.

We calculated the geometric mean of the unit risk factors from Table 3 using Equation 3. We applied the geometric mean instead of the arithmetic mean to reduce the influence of extreme values in our small sample of unit risk estimates.

Equation 3: Geometric mean

$$GeomMean = e^{\frac{\sum_{i=1}^{n} \ln(UR_i)}{n}}$$

GeomMean = Geometric mean

i = Considered source with unit risk data

n = Number of considered sources

UR_i = Unit risk factors according to source i

The 95% confidence interval of the geometric mean unit risk was estimated assuming a t distribution and applying Equation 4.

Equation 4: Confidence interval for average relative risk

$$CI = e^{\frac{\sum_{i=1}^{n} \ln(UR_i)}{n} \pm t_{n-1} * \frac{sd(\ln(UR_1)...\ln(UR_n))}{\sqrt{n}}}$$

CI = Confidence interval (lower and upper bound)

UR_i = Unit risk factors according to source i

sd = standard deviation

n = sample size

Results

Equivalent concentration

Table 4 shows the PM_{10} equivalent increase in concentration (difference between exposure scenario and counterfactual concentration), which would cause the number of lung cancer deaths of the three risk levels (1 in 10,000, 1 in 100,000, and 1 in 1,000,000) and of the related toxicology-based risk scenarios C2, C3 and C4 (5 in 10,000, 5 in 100,000 and 5 in 1,000,000, respectively).

Table 4: Estimated PM_{10} concentration causing one and five deaths, respectively, due to lung cancer, for the three risk scenario levels in Switzerland.

Number of pollutants	Accepted risk level (in lifetime lung cancer deaths)	Increase in population-weighted PM_{10} annual mean concentration in μg/m^3 using the epidemiolocal approach (lower & upper bound)[a]
One pollutant	1 in 10,000	0.333 (0.866; 0.226)
	1 in 100,000	0.033 (0.087; 0.023)
	1 in 1,000,000	0.003 (0.009; 0.002)
Five pollutants	5 in 10,000 (scenario C2)	1.664 (4.332; 1.13)
	5 in 100,000 (scenario C3)	0.166 (0.433; 0.113)
	5 in 1,000,000 (scenario C4)	0.017 (0.043; 0.011)

[a] The lower and upper bound were derived using the available 95% confidence interval of the relative risk.

Sensitivity analysis

In the sensitivity analysis (Table 5) we increased the value of the input data by 10%. The sensitivity analysis is restricted to the scenarios A2 and C1, i.e. counterfactual concentration in 2010 for PM_{10} and for the five carcinogens as well as for the counterfactual concentration of 7.5 μg/m^3 for PM_{10}. The epidemiological approach appeared more sensitive to the 10% increase of the input values than the toxicological approach. The highest sensitivity was found when changing the value of the counterfactual PM_{10} concentration. Increasing this value by 10%, results in a 17.1% higher number of PM_{10} attributed cases. For the toxicological approach, increasing by 10% the counterfactual concentration of carcinogens, the population or the unit risk factors increases the number of deaths by 10%.

Table 5 Sensitivity analysis showing the change in the number of attributable lung cancer deaths in Switzerland if the values of the input variables used in the epidemiological and toxicological approach increase by 10% for the scenario "exposure data from 2010".

Approach	Variable	Basis value	Value after increasing 10%	Change in terms of annual lung cancer deaths per 100,000persons (%) if the basis value of the variable increases by 10%
Epidemiological (scenario A2)	Ambient population-weighted mean concentration of PM_{10} exposure scenarios in µg/ m^3	18 µg/m^3	19.8 µg/m^3	17.1%
	New lung cancer cases per year	4,300	4,730	10%
	Relative risk	1.08	1.088	9.7 %
	Ambient counterfactual population-weighted mean concentration PM_{10} in µg/m^3	7.5 µg/m^3	8.3 µg/m^3	-7.1%
Toxicological (scenario C1)	Ambient population-weighted mean concentration of exposure scenarios for five carcinogens in µg/ m^3	From $1.2x10^{-4}$ to $9.4x10^{-1}$ µg/m^3	From $1.3x10^{-4}$ to $1.0x10^{-1}$ µg/m^3	10%
	Population aged 30 and older	5,663,968	6,230,365	10%
	Unit risk	From 0. 429 to 3.959 lung annual cancer cases per 100 000 persons and 1 µg/m³ carcinogen	From 0.472 to 4.355 lung annual cancer cases per 100 000 persons and 1 µg/m³ carcinogen	9.8%
	Years of lifetime	70	77	-9.1%

Comparison of relative risk estimates

Particularly, relative risk estimates can widely range depending on the published meta-analysis, the geographical focus, the PM type (PM_{10} vs. $PM_{2.5}$) and the health effect (mortality vs. incidence). Therefore, we additionally compared the health burdens of nine relative risk estimates, which were identified in the literature (the one selected for this study plus eight additional ones). We found that the number of annual lung cancer deaths for the scenario A2 ranges from 98 to 1,079. Table 6 shows the different number of lung cancer deaths that are estimated when using the different available relative risk estimates.

Table 6 Estimated yearly lung cancer deaths of the identified relative risk estimates in Switzerland for the scenario “exposure data from 2010”.

Relative risk estimate (95% confidence interval)[a]	Source	PM type	Health effect	Geographic scale	Yearly lung cancer deaths in Switzerland for scenario A2 (lower bound; upper bound)[b]
1.08 (1.03; 1.12)[c]	Huang et al. (2017)	$PM_{2.5}$	Incidence	World	255 (98; 376)
1.03 (0.61; 1.75)	Huang et al. (2017)	$PM_{2.5}$	Incidence	Europe	98 (-1640; 1,856)
1.11 (1.05; 1.18)	Huang et al. (2017)	$PM_{2.5}$	Mortality	World	346 (162; 549)
1.05 (1.01; 1.10)	Huang et al. (2017)	$PM_{2.5}$	Mortality	Europe	162 (33; 316)
1.08 (1; 1.17)	Hamra et al. (2014)	PM_{10}	Incidence & mortality	World	347 (0; 709)
1.09 (1.04; 1.14)	Hamra et al. (2014)	$PM_{2.5}$	Incidence & mortality	World	286 (130; 435)
1.27 (0.96; 1.68)	Hamra et al. (2014)	PM_{10}	Incidence & mortality	Europe	1,079 (-184; 2,342)
1.03 (0.89; 1.2)	Hamra et al. (2014)	$PM_{2.5}$	Incidence & mortality	Europe	98 (-387; 605)
1.22 (1.03; 1.45)	Raaschou-Nielsen et al. (2013)	PM_{10}	Incidence	Europe	898 (133; 1,678)

[a] Estimate per 10 µg/m3 increase of PM.

[b] Scenario A2 means PM_{10} exposure data from 2010 and 7.5 µg/m3 as counterfactual population-weighted concentration (see published paper). Lower and upper bound are estimates based on the lower and upper bound of the confidence interval of the relative risk.

[c] Relative risk used in our study.

Discussion

Comparison of results with a previous study

In a former assessment for the city of Basel (Röösli et al. 2003), the lung cancer burden attributed to PM_{10} for a concentration difference of 17.5 µg/m^3 was found to be 12 times higher than the one assigned to 13 carcinogens. In our study we found that the lung cancer burden of a PM_{10} difference of 14.7 µg/m^3 (i.e. the exposure scenario of 18 µg/m^3 versus the counterfactual concentration of 3.3 µg/m^3 in the scenario B2) is about 14 times higher than the one of the five carcinogens, which is a similar result..

Furthermore, some of the carcinogens are markers of larger groups of substances. For example, elemental carbon is a marker of a group of substances in diesel exhaust and BaP is a marker of a group of polycyclic aromatic hydrocarbons (PAHs). BaP is only one of about 50 different PAHs generated by wood burning (Bruns et al. 2015). Samburova et al (2017) concluded that 16 particle-bound PAHs explained only 15.4% of the BaP-equivalent toxicity derived for 88 gas- and particle-

bound PAHs. Röösli et al (2003) included PAHs as a group of substances in the risk assessment of the city of Basel. However, PAH contributed only 4.1% to the burden of the 14 carcinogens included in their assessment (BaP alone contributed 8% in our study). On the one hand, considering the whole group of substances of diesel exhaust (instead of elemental carbon as marker) and all PAHs (instead of only BaP as marker) would imply a higher health burden. On the other hand, a part of PAHs are included in diesel exhaust. Therefore, considering diesel exhaust and all PAHs would lead to an overlap.

References

BAFU. 2019. Air pollution concentration data for Switzerland. E-mail communication with Rudolf Weber (Federal Office for the Environment, BAFU in German). Unpublished work.

BFS. 2017. Ständige Wohnbevölkerung nach Alter, Geschlecht und Staatsangehörigkeitskategorie, 2010-2016. Swiss Fed Stat Off BFS Ger. Available: https://www.bfs.admin.ch/bfs/en/home/statistics/catalogues-databases/tables.assetdetail.3202895.html [accessed 2 October 2018].

Bruns EA, Krapf M, Orasche J, Huang Y, Zimmermann R, Drinovec L, et al. 2015. Characterization of primary and secondary wood combustion products generated under different burner loads. Atmospheric Chem Phys 15:2825–2841; doi:https://doi.org/10.5194/acp-15-2825-2015.

Hamra GB, Guha N, Cohen A, Laden F, Raaschou-Nielsen O, Samet JM, et al. 2014. Outdoor particulate matter exposure and lung cancer: a systematic review and meta-analysis. Environ Health Perspect 122:906–911; doi:10.1289/ehp/1408092.

Huang F, Pan B, Wu J, Chen E, Chen L. 2017. Relationship between exposure to PM2.5 and lung cancer incidence and mortality: A meta-analysis. Oncotarget 8:43322–43331; doi:10.18632/oncotarget.17313.

IARC-WHO. 2018. IARC Monographs on the Identification of Carcinogenic Hazards to Humans. Web Portal Int Agency Cancer Res World Health Organ Cancer Epidemiol Genet Databases. Available: https://monographs.iarc.fr/list-of-classifications-volumes/ [accessed 7 February 2019].

Krebsliga Schweiz. 2018. Krebs in der Schweiz: wichtige Zahlen.

NIH. 2019. PubChem: open chemistry database.

OEHHA. 2009. Technical Support Document for Cancer Potency Factors: Methodologies for derivation, listing of available values, and adjustments to allow for early life stage exposures.

Raaschou-Nielsen O, Andersen ZJ, Beelen R, Samoli E, Stafoggia M, Weinmayr G, et al. 2013. Air pollution and lung cancer incidence in 17 European cohorts: prospective analyses from the European Study of Cohorts for Air Pollution Effects (ESCAPE). Lancet Oncol 14:813–822; doi:10.1016/S1470-2045(13)70279-1.

Röösli M, Künzli N, Schindler C, Theis G, Oglesby L, Mathys P, et al. 2003. Single Pollutant Versus Surrogate Measure Approaches: Do Single Pollutant Risk Assessments Underestimate the Impact of Air Pollution on Lung Cancer Risk? J Occup Environ Med 45:715–723; doi:10.1097/01.jom.0000079082.33909.c2.

Samburova V, Zielinska B, Khlystov A. 2017. Do 16 Polycyclic Aromatic Hydrocarbons Represent PAH Air Toxicity? Toxics 5; doi:10.3390/toxics5030017.

Swiss Federal Council. 2018. Ordinance on Air Pollution Control (OAPC) of 16 December 1985 (Status as of 1 June 2018) CC 814.318.142.1.

Swiss Federal Council. 1997. Ordinance on the Incentive Tax on Volatile Organic Compounds.

USEPA. 2013. Integrated Risk Information System. Available: https://www.epa.gov/iris [accessed 10 July 2018].

WHO-Europe. 2000. Air Quality Guidelines for Europe.
